# Culturally enhancing a group-based motivational interviewing substance use prevention program for Latine youth^☆^

**DOI:** 10.1016/j.conctc.2022.100991

**Published:** 2022-09-11

**Authors:** Oswaldo Moreno, Melissa Avila, Isis Garcia-Rodriguez, Stephanie Romo, Jennifer Rodriguez, Cristian Matos, Lisa S. Fuentes, Cindy Hernandez, Mayra S. Ramos, Geovani Muñoz, Daniel Gutierrez, Adrian J. Bravo, Rosalie Corona

**Affiliations:** a Virginia Commonwealth University, United States; b The College of William & Mary, United States; c Georgetown University, United States

**Keywords:** Motivational interviewing, Substance use, Alternative tobacco products, Latine, Youth

## Abstract

**Background:**

Group Motivational Interviewing for Teens (GMIT) has been effective in reducing youth substance use in diverse communities, yet more research is needed to determine its efficacy in reducing tobacco and alternative tobacco products (ATP) use among Latine adolescents. This study modified GMIT to include a focus on ATPs (GMIT-ATP). GMIT was also linguistically translated so it could be offered in English and Spanish, culturally enhanced, and parent sessions were added (GMIT-ATP + P).

**Methods:**

The study's aims were to 1) Develop a model of how cultural context, family relationships, and adolescent tobacco-related skills/beliefs are associated with smoking and ATP use; 2) Examine the impact of the GMIT-ATP intervention on adolescent tobacco use; 3) Examine whether the GMIT-ATP + P intervention improves family/parenting factors associated with reduced adolescent tobacco use; 4) Examine whether GMIT-ATP + P is more effective than GMIT-ATP in improving adolescent tobacco use; 5) Explore whether essential components of our behavior change model mediate the impact on tobacco use, and 6) Explore whether cultural factors influence the impacts of our intervention. Latine adolescents (ages 10–16) and their parents/guardians were recruited throughout Virginia. Parents and adolescents completed three surveys: before and immediately after the program ends and at 3-months post-intervention. Families attended 3 GMIT-ATP or GMIT-ATP + P sessions.

**Conclusion:**

Findings from this study will be disseminated in Latine communities and with providers working with Latine youth and can serve as a community-based model to reduce substance and tobacco use (e.g., ATP) in these Latine communities.

The Latine population will comprise nearly 30% of the total U.S. population by 2060 [1]. Given the rise of the Latine population, more prevention efforts are needed to address Latine substance use needs, particularly tobacco use. For example, Latine smokers are less likely than non-Latines to seek prevention services around smoking, be screened for smoking, and engage in evidence-based cessation treatments [2,3]. Latine youth specifically may need additional tobacco prevention efforts for several reasons. First, data suggest that Latine youth are increasing substance and tobacco use onset, including alternative tobacco products (ATPs). For example, youth's onset for smoking and frequency (smoking more than ten cigarettes per day) was the highest compared to their non-Latine peers [4]. Regarding ATPs (like e-cigarette use), [5]; using the 2020 National Youth Tobacco Survey (NYTS), found that 7.1% of Latine youth had used ATPs (e.g., e-cigarettes) in the past month, compared with only 4.3% of non-Hispanic whites; this suggests that Latine youth may be more likely than non-Latine whites to initiate ATP use. Second, Latine youth consistently report higher substance and ATP use susceptibility levels than other racial/ethnic groups [6]. Third, Latine youth are more likely to develop a substance use disorder in the future due to their early onset [7,8]. Fourth, as Latine youth transition to adulthood, they are disproportionately burdened by the health consequences of substance and tobacco use (e.g., cancer, heart disease; [9,10]. Although efforts are striving to prevent the onset of and frequency of substance and tobacco use for Latine youth, more attention is needed for culturally enhancing prevention interventions. For example, although the Group Motivational Interviewing for Teens (GMIT) has effectively reduced youth substance use in diverse communities [11,12], more research is needed to determine its efficacy in reducing tobacco and alternative tobacco products (ATP) use in a culturally relevant fashion among Latine adolescents. This study, therefore, modified GMIT to include a focus on ATPs (GMIT-ATP). GMIT was also linguistically translated so it could be offered in English and Spanish and culturally enhanced in ways that included parent sessions (GMIT-ATP + P).

## 1 Incorporating culture into tobacco prevention

Family plays a vital role in the Latine culture, evidenced by several cultural values emphasizing family relationships. For example, *familismo*, conceptualized as the value of family as the primary source of social support and identity [13,14], highlights intimate family bonds built on loyalty and solidarity throughout one's life [15]. Specifically, higher levels of *familismo* are associated with decreased Latine adolescent tobacco use [16,17]. *Familismo* can also impact Latine adolescents' intervention engagement [18]. One study found that Latine adolescents' engagement in a substance use prevention intervention differed based on the adolescents' cultural values and ethnic identity. Specifically, Latine adolescents with low ethnic identity and *familismo* responded best to the standard intervention, whereas Latine adolescents with higher ethnic identity and *familismo* responded best to the culturally accommodated intervention.

Stein and colleagues (2014) conceptualized parenting practices such as parental monitoring and close/warm family relationships as behavioral manifestations of *familismo* [19]. Robust findings in the literature are that increased parental monitoring and close/warm relationships are associated with decreased tobacco use among Latine adolescents [20,21]. Engaging parents in tobacco use prevention for Latine adolescents is important and culturally relevant. Research has demonstrated the efficacy of parenting programs in reducing Latine adolescent risk behaviors, including tobacco use, and promoting better parent-child relationships (e.g., [22,23]).

Engaging parents in prevention programs remains a significant barrier despite the importance and cultural relevance of including parents. Extant family interventions for Latines are relatively long (usually around 10-17-week sessions). Brief interventions may be particularly suitable for Latines given their high rates of underutilization treatment, high dropout rates, and the multiple practical and cultural barriers they experience when accessing substance use services [24,25]. Further, parent groups usually suffer low attendance and retention rates [24,26]. These low attendance rates may result from parents' busy work schedules, transportation, childcare issues, and adolescents’ extracurricular activity schedules. Therefore, brief interventions may be the most feasible approach to engage families whose adolescents are in the earlier stages of tobacco use or whose parents do not believe they are using tobacco. Further, culturally relevant substance and tobacco prevention programs incorporating the recent trends of ATPs and engaging parents are limited, especially for Latine youth.

## 2 Using motivational interviewing to prevent adolescent tobacco use

Motivational Interviewing (MI), an approach designed to increase motivation towards change [27], has shown health-promoting effects. MI is a client-centered, collaborative, and non-judgmental approach [27] that integrates cultural values as essential elements of the individual's narrative [28]. Several meta-analyses have documented strong empirical support for the efficacy of MI in adults in addressing various issues, including alcohol and drug use, tobacco use, and risky behaviors [29,30]. Furthermore, MI effectively improves an individual's session attendance (e.g., fewer dropouts and missed sessions) when implemented in a university-based clinical setting [31].

MI interventions have also reduced youth alcohol and drug use [32,33] and tobacco use [34,35]. Borelli and colleagues (2015) further note strong support for providing MI as part of parent-child health interventions [36]. Using a 3-session MI intervention with adolescents and parents facilitated their engagement with a prevention program focused on adolescent problem behaviors which in turn was associated with decreased teenage substance use [37,38]. Finally, Anez and colleagues (2008) found MI congruent with core Latine cultural values, highlighting its relevance to working with Latine adolescents and their families [39].

## 3 Group Motivational Interviewing for Teens

The main base of our intervention framework is a group MI intervention developed by D'Amico and colleagues that is available online at no cost (www.groupmiforteens.org/about) (GMIT; [11,12,32,40]. A cluster randomized controlled trial (*n* = 9528) revealed a school-wide effect where youth in schools receiving the MI intervention were less likely to initiate alcohol use than youth not receiving the MI treatment [32]. Previous pilot studies with GMIT also demonstrated that youth selected to attend came from ethnically diverse backgrounds (e.g., Native Americans, Asians, Hispanic/Latine, African American, multiracial, etc.) who typically seek fewer services. Although these findings suggest favorable outcomes when using a group MI approach with adolescents at-risk for drinking, there is still a need for research to address tobacco use, including ATPs, with Latine (especially Spanish-speaking) youth.

## 4 Integrating ATP and cultural enhancements into GMIT

We integrated a module on ATP developed by the Virginia Foundation for Healthy Youth (Nicotine Products Prevention Modules [formerly known as Other Tobacco Products Modules], 2022) into GMIT to create a comprehensive tobacco use prevention program (GMIT-ATP). We also culturally enhanced GMIT-ATP because interventions adapted to focus on a specific cultural group are four times more effective than those focused on the general population, and interventions provided in an individual's native language are twice as effective [41]. Specifically, we linguistically translated the GMIT-ATP intervention and addressed other surface-level enhancements (e.g., changing pictures). Given the importance of the cultural value of *familismo*, we also integrated a parent component (GMIT-ATP + P).

## 5 Participant recruitment

Participants were able to participate in the study if they identified as Latine youth between 10 and 16 years of age. Given that some middle school students report using tobacco and other substances, it is possible that the adolescents who participated in this study were currently using some substances or have used substances in the past. Therefore, Latine youth who reported current and/or prior substance use (but were not previously diagnosed with a substance use disorder) were allowed to participate in the study. Siblings in the same family dyad between 10 and 16 were also allowed to participate.

The intervention was implemented at the largest local Latine community center, with adequate space to see multiple families. We recruited from the local community with the help of this local Latine community center and other community partners. However, we also transitioned to an online intervention modality during the COVID-19 pandemic, at which point Latine families throughout Virginia were encouraged to participate in the intervention. Study flyers gave families a number to call to receive more information about the study. When families called, the research staff read a detailed description of the study to families (i.e., an IRB-approved phone script). Families were asked if they were interested in participating. If they were interested in participating, families scheduled an interview with the research team to consent and assent to the research and collect baseline data. Once the baseline data was completed, families were randomized to either group. During the pandemic, randomization was broken due to recruitment and COVID-19 challenges. Families were assigned to a group so the group could be implemented as quickly as possible. The University IRB approved all in-person-to-online changes made to the intervention.

## 6 Pilot trial

We conducted a pilot study of the GMIT-ATP (n = 40 families) and GMIT-ATP + P (n = 40 families) interventions. These interventions were facilitated by advanced bilingual Latine doctoral students with clinical training. Specifically, we aimed to enroll eight families per group (e.g., eight families X 5 groups = 40 participants per condition). The sessions and session length were equivalent across the two conditions. All adolescents received the GMIT-ATP intervention; some parents received the GMIT-ATP + P session while others did not. Parents and adolescents were not informed that one of the two interventions included sessions focused on parenting strategies. According to our recommended IRB procedures, the purpose of the study was explained to families after they completed the final post-evaluation measures. This was the ideal balance between a methodologically sound design and adequately informed participants.

Parents and adolescents in the intervention and control groups completed measures (see Table 1) before the program started, immediately after program completion, and 3-months after program completion. Depending on the participant's language preferences and abilities, groups were held in Spanish or English. Program material and examples were explained or clarified in English or Spanish, and youth were encouraged to ask for clarification in either language to improve comprehension. End-of-session rating forms were completed online through Google Forms, and baseline, post-intervention, and 3-month follow-up assessment surveys were completed online via Redcap.

**Table 1:** Assessment measures and timeline.

Measure	Brief Description	Example Item and Response Scale	Baseline	Post-Intervention	3-month Follow-Up
*Measures Completed by Parents and Adolescents*					
Demographics – Inclusive (Hughes et al., 2016)	General demographic questions were asked in addition to including more inclusive answer choices meant to reflect the complexity of respondents' identities. Two versions of this measure were created (one for youth and one for parents) since certain questions were directed towards parents only (e.g., total family income).	“What language is currently spoken in your home?” Response scale: (1) only English, (2) mostly English, (3) English and Spanish equally, (4) mostly Spanish, (5) only Spanish, (6) other	X	X	X
Mexican American Cultural Values Scale (MCVS; Knight et al., 2010)	The MCVS measures the degree to which someone endorses values/beliefs that are more associated with Mexicans/Mexican Americans. The Latine subscales of interest are: familismo, respect, religion, and traditional gender roles.	“Children should be taught that it is their duty to care for their parents when their parents get old.” Response scale: (1) not at all, (2) a little, (3) somewhat, (4) very much, (5) completely	X	X	X
Multiphasic Assessment of Cultural Constructs-Short Form (MACC-SF; Cuéllar et al., 1995)	The MACC-SF measures cultural factors that are common across Hispanic cultures. The cultural subscales of interest are: Fatalismo and personalismo subscales were completed.	“It doesn't do any good to try to change the future because the future is in the hands of God.” Response scale: (0) false, (1) true	X	X	X
Bidimensional Acculturation Scale (BAS; Marin & Gamba, 1996)	The BAS is used to measure changes in language-related behaviors (general use, proficiency, and use in media) in Hispanic and non-Hispanic cultural domains.	“How often do you speak in English with your friends?” Response scale: (1) almost never, (2) sometimes, (3) often, (4) almost always; for items 1–6 and 19-24 (1) very poorly, (2) poorly, (3) well, (4) very well; for items 7-18	X	X	X
Acculturative Family Distancing scale (AFD; Hwang et al., 2010)	The AFD measures the distancing between a parent and a child that results from immigration, cultural differences, and differing rates of acculturation.	“I share personal things with my parent(s)/child.” Response scale: (1) strongly disagree, (2) moderately disagree, (3) mildly disagree, (4) neither agree nor disagree, (5) mildly agree, (6) moderately agree, (7) strongly agree	X	X	X
Parenting Practices Scale (PPS; Stattin et al., 2000)	The PPS assesses the behaviors of both parents and children that relate to parents' awareness of their children's activities. The response scale varies greatly depending on the question.	“Does your parent/you know what you/your child do(es) during your/their free time?” Response scale: (1) Almost always, (2) Usually, (3) It depends, (4) Seldom, (5) Never	X	X	X
Network of Relationships Inventory-Relationships Quality Version—Conflict (NRIRVQ-C; Buhrmester & Furman, 2008)	The NRIRVQ assesses 10 subscales of relationship qualities to describe the supportive and discordant qualities of relationships among children, adolescents, and adults. However, only the conflict subscale was measured in this study.	“How often do you and your parent disagree and quarrel with each other?” Response scale: (1) never or hardly at all, (2) seldom or not too much, (3) sometimes or somewhat, (4) often or very much, (5) always or extremely much	X	X	X
Family Adaptability & Cohesion Evaluation Scale III (FACES-III; Olson et al., 1985)	This 10-item version of the FACES-III cohesions subscale assessed parent and adolescent perceptions of how often family members do various behaviors representing family cohesion.	“When our family gets together for activities, everybody is present.” Response scale: (1) almost never, (2) once in a while, (3) sometimes, (4) frequently, (5) almost always	X	X	X
Parent-Adolescent Communication Scale (PACS; Barnes et al., 1985)	The PACS is a 20-item measure comprised of two subscales that measure the degree of openness and extent of problems in family communication.	“I can discuss my beliefs with my parent without feeling restrained or embarrassed.” Response scale: (1) Strongly disagree, (2) Disagree, (3) Undecided, (4) Agree, (5) Strongly agree	X	X	X
Parental Messages about Substance Use (PMSU; Jackson, & Henriksen, 1997)^a^	The PMSU measures parent child communication about substance use by asking youth to complete the six-item questionnaire that was modified to include additional forms of substance use beyond tobacco use.	How many times in the last 6 months have you talked with your parent about the following topics related to alcohol use? “Negative consequences of alcohol use.” Response scale: (0) 0 times, (1) 1 time, (2) 2 times, (3) 3 or more times	X	X	X
Perceived Stress Scale (PSS; Cohen & Williamson, 1988)	The PSS evaluates the degree to which an individual has perceived life as unpredictable, uncontrollable, and overloading over the previous month.	“In the last month, how often have you been upset because of something that happened unexpectedly?” Response scale: (0) never, (1) almost never, (2) sometimes, (3) Fairly often (4) Very Often	X	X	X
Duke University Religion Index (DUREL; Koenig et al., 1997)	The DUREL is a 5-item measure of religious involvement. The instrument assesses three major dimensions of religiosity: organizational religious activity, non-organizational religious activity, and intrinsic religiosity (or subjective religiosity).	“How often do you attend church, synagogue, or other religious meetings? Response scale: (0) never, (1) almost never, (2) sometimes, (3) Fairly often (4) Very Often	X	X	X
Daily Spiritual Experience Scale (DSES; Underwood, 2011)	The DSES assesses ordinary experiences of connection with the transcendent in daily life. It includes constructs such as awe, gratitude, mercy, sense of connection with the transcendent and compassionate love	“I feel God's presence.” Response scale: (1) Many times a day, (2) every day, (3) most days, (4) some days, (5) Once in a while, (6) never/almost never	X	X	X
Everyday Discrimination Scale – Racial and Weight (Williams et al., 1997)	This adapted version of the day-to-day unfair treatment scale asks individuals to rate how often certain discriminatory experiences have happen to them because of their race, ethnicity, or color as well as their weight (i.e., perceived discrimination).	“You are treated with less courtesy than other people are.” Response scale: (0) never, (1) less than once a year, (2) A few times a year, (3) A few times a month, (4) at least once a week, (5) Almost everyday	X	X	X
Strengths and Difficulties Questionnaire (SDQ; Goodman, 1997)	The SDQ is a measure of youth psychopathology that yields a total score and five subscale scores: emotion problems, conduct problems, hyperactivity, peer problems and prosocial.	“I am easily distracted, I find it difficult to concentrate.” Response scale: (0) Not true, (1) Somewhat true, (3) Certainty true	X	X	X
*Youth Only Measures*					
Multi-group Ethnic Identity Measure (MEIM; Huang & Stormshak, 2011, Roberts et al., 1999)	The MEIM is used to examine an individual's sense of belonging to their ethnic group, one's attitudes about the group, and identification with it. Eight items used with younger adolescents were added to the original 12-item version.	“I have spent time trying to find out more about my own ethnic group, such as its history, traditions, and customs.” Response scale: (1) strongly disagree, (2) disagree, (3) agree, (4) strongly agree	X	X	X
Bicultural Stress Scale (BSS; Romero et al., 2003)	The BSS is used to measure perceived stress within a bicultural context through questions about everyday stressors (e.g., discrimination, intragroup pressures, intergroup conflict, and acculturation) within schools, peers, and family contexts. addresses everyday stressors within schools, peers, and family contexts, and include factors related to discrimination, intragroup pressures (Rodriguez et al., 2002; Romero & Roberts 2003a), intergroup conflict, and acculturation	“Because of family obligations I can't always do what I want.” Response scale: (1) not at all stress, (2) a little bit stressful, (3) quite a bit stressful, (4) very stressful, (5) does not apply	X	X	X
Problem Behavior Frequency Scale-Substance Use (PBFS-SU; Farrell et al., 2000; Kandel 1975)^a^	The substance use version of the PBFS measures the frequency of drug and alcohol use in the past month.	In the last 30 days, how many times have you done the following: “Drunk liquor (like whiskey or vodka)” Response scale: (0) never, (1) 1–2 times, (2) 3–5 times, (3) 6–9 times, (4) 10–19 times, (5) 20 or more times	X	X	X
Alternative Tobacco Product Survey (Other Tobacco Products Modules; VFHY, 2019)	This pre-test survey developed by the Virginia Foundation for Healthy Youth measures lifetime and past month tobacco use as well as intention to use tobacco in the future. A second part with nine questions assesses knowledge and awareness of tobacco products.	“Which of the following products have you ever tried?” Response scale: Cigarettes (e.g., Marlboro), Cigars/Cigarillos (e.g., Black & Mild), Electronic cigarettes (e.g., JUUL), Hookah Smokeless tobacco products, (e.g., Skoal), Other, None	X	X	X
Tobacco Related Knowledge, Attitudes, Beliefs, And Norms (TKABNS; Primack et al., 2007)^a^	The TKABNS assesses beliefs and attitudes associated with tobacco related products.	“Do you think young people who smoke cigarettes have more friends?” Response scale: (1) Definitely yes, (2) Probably yes, (3) Probably not, (4) Definitely not	X	X	X
Smoking Future Expectations Scale (SFES, Pierce et al., 1998)^a^	The SFES considers a person as “non-susceptible” if the person answers “definitely no” to whether or not they believe they will smoke soon, in the next year, and if a friend offers a cigarette. This study also asked about experimenting with cigarettes in the future.	“Do you think that you will smoke a cigarette soon?” Response scale: (1) Definitely yes, (2) Probably yes, (3) Probably not, (4) Definitely not	X	X	X
Smoking Normative Beliefs (SNB; Primack et al., 2007)^a^	The SNB measures beliefs centered around perceived prevalence, perceived disapproval, and likelihood that certain people are smokers.	“Most successful businesspeople smoke cigarettes at least once a month.” Response scale: (1) strongly disagree, (2) disagree, (3) agree, (4) strongly agree for items 1–7 and 0–100% in increments of 10% for items 8-11	X	X	X
Refusal Intentions Scale (RIS; Redmond et al., 2009^a^	Refusal Intention for offers of specific substances; Intentions regarding substance resistance strategies.	How likely are you to handle these situations in this way: “Smoke a cigarette?” Response scale: (1) Definitely would say “no”, (2) probably would say “no”, (3) not sure, (4) probably would not say “no”, (5) definitely would not say “no”	X	X	X
Tobacco and Alcohol Use Refusal Efficacy^a^ (TAURE; Conners et al., 2003)	The TAURE assesses one's ability to resist drug use under various specific circumstances. This measure was modified to include ATPs.	“If I thought that my friends would like me more if I did it.” Response options: (0) Yes, (1) Not sure, (2) No	X	X	X
Revised Children's Anxiety and Depression Scale (RCADS-25; Ebesutani et al., 2012)	The RCADS-25 assesses depression and anxiety in children and adolescents.	“I feel sad or empty.” Response scale: (0) never, (1) sometimes, (2) always, (3) often	X	X	X
General Pet Questions (PETS; Hawkins & Williams, 2017)	The PETS measure is adapted from the Childhood Pet Ownership Questionnaire (Paul & Serpell, 1993) and asks about current and past ownership of pets, types of pets, the number of pets in the household, and whether there was a pet that the child considered to be their own.	“In your in your LIFETIME have you ever had a pet?” Response scale: (0) No, (1) Yes	X	X	X
Pet Attachment and Life Impact Scale (PALS-35; Cromer & Barlow, 2013)	The PALS assesses the attachment to pets, positive and negative aspects of relationships with pets and the impact of pets on their owners.	“Having a Pet has helped my health.” Response scale: (1) Not at all, (2) somewhat, (3) moderately, (4) quite a bit, (5) very much	X	X	X
Program Satisfaction Survey - Adolescent	The satisfaction survey asked the youth to indicate their level of agreement or disagreement with specific statements regarding what they may have enjoyed or found useful (or not) about the intervention. They could also provide free response suggestions if they desired.	“I have learned skills to help me make the decision not to use tobacco” Response scale: (SA) strongly agree, (A) agree, (N) neutral, (D) disagree, (SD) strongly disagree		X	X
*Parent Only Measures*					
Multidimensional Acculturative Stress Inventory (MASI; Rodriguez et al., 2015	Traditionally, the MASI was developed to assess acculturative stress among individuals of Hispanic or Mexican origin in the US through 36 questions.	If you have experienced this situation during the past 3 months, how stressful has each situation been? “I don't speak English (Spanish) or don't speak it well” Response scale: (1) yes, (2) no If yes, then (1) not at all stressful, (2) slightly stressful, (3) somewhat stressful, (4) moderately stressful, (5) extremely stressful	X	X	X
Substance Use (2010 National Survey on Drug Use and Health)	This set of four questions taken from the 2010 National Survey on Drug Use and Health survey ask parents about their lifetime and recent alcohol, cannabis, and recreational prescription medication use.	“Have you ever, even once, used marijuana or hashish?” Response scale: (1) yes, (0) no	X	X	X
Tobacco Use (2010 National Survey on Drug Use and Health)	This set of six questions taken from the 2010 National Survey on Drug Use and Health survey ask parents about their lifetime and recent tobacco use and that of any other members in the household.	“Have ever used any of the following tobacco products? Cigarettes, cigars, electronic cigarettes, hookah, smokeless tobacco, other” Response scale: (1) yes, (0) no	X	X	X
Generalized Anxiety Disorder (GAD-7; Spitzer et al., 1999, 2006)	The GAD-7 is a self-report measure used to assess anxiety. Participants are queried regarding symptoms during the past two weeks.	Over the last 2 weeks, how often have you been bother by the following problems? “Trouble Relaxing” Response scale: (0) not at all (1) several days (2) more than half of the days, (3) nearly every day	X	X	X
Patient Health Questionnaire (PHQ-9; Kroenke et al., 2001, Spitzer et al., 1999)	The PHQ-9 assesses the degree of depression present in an individual over the last two weeks.	Over the last 2 weeks, how often have you been bother by the following problems? “Poor Appetite or overeating” Response scale: (0) not at all (1) several days (2) more than half of the days, (3) nearly every day	X	X	X
Barriers to access to care evaluation scale (BACE v2; Clement et al., 2012)	The BACE v2 measures various reasons that may have delayed, stopped, or discouraged a respondent from seeking professional care for a mental health problem.	Have any of these issues ever stopped, delayed, or discouraged you from getting, or continuing with, professional care for a mental health problem? “Concern that I might be seen as a bad parent” Response scale: (0) not a lot, (1) a little, (2) quite a lot, (3) a lot	X	X	X
Mental Help Seeking Attitudes Scale (MHSAS; Hammer et al., 2018)	The MHSAS measures respondents' overall evaluation (unfavorable vs. favorable) of their seeking help from a mental health professional if they found themselves to be dealing with a mental health concern.	If I had a mental health concern, seeking help from a mental health professional would be … “Useless 3 2 1 0 1 2 3 Useful” Response scale: 3 to 3, participant circles the number closest to the response that best represents their belief	X	X	X
Cultural Humility Scale (CHS; Hook et al., 2013)^2^	The CHS asks people to identify the aspect of their cultural background that is most central or important to them as well as the perceived cultural humility of their therapist. The word therapist was replaced with group facilitator to assess the parents perceived cultural humility of the parent support group facilitator.	“Please identify the aspect of your cultural background that is most central or important to you” “How important is this aspect of your cultural background?” Response: Free Response following by 1 (not at all important), 2, 3(somewhat important, 4, or 5 (very important)	X	X	X
Cultural (missed) Opportunities Scale (CmOS; Owen et al., 2016)	This CmOS measures whether clients feel that their therapist adequately addressed their cultural background within therapy sessions. The word therapist was replaced with group facilitator to assess whether parents felt the facilitator adequate addressed their cultural background in the intervention parent support group sessions. Only parents who were assigned the GMIT-ATP + P completed this measure.	“My group leader discussed my cultural background in a way that worked for me” 5-point Likert scale: (1) strongly disagree to (5) strongly agree		X	X
Fear of Deportation (FoD; Arbona et al., 2010)	The FOD discerns if the respondent avoided seeking government services, attending court, reporting crimes done onto others or oneself, or being in public for fear of deportation	“Have you ever avoided or not walked in the streets because of fear or concern of being deported?” Response scale: (0) no, (1) yes	X	X	X
Program Satisfaction Survey-Parent	The satisfaction survey asked the parents to indicate their level of agreement or disagreement with specific statements regarding what they may have enjoyed or found useful (or not) about the intervention. They could also provide free response suggestions if they desired. Parents who were assigned to the GMIT-ATP + P completed an alternate version of this survey that included two additional questions about the parent group facilitator.	“I would recommend the intervention to other parents” Response scale: (SA) strongly agree, (A) agree, (N) neutral, (D) disagree, (SD) strongly disagree		X	X

a In these measures, additional question(s) were added to the original measure to assess participant's beliefs or attitudes towards or use of alternative tobacco products, including e-cigarettes.

## 7 Summary of sessions and cultural enhancements

Table 2 summarizes the sessions’ cultural enhancements. Given the GMIT-ATP + P was developed as means to culturally enhance the GMIT-ATP protocol, parents in the GMIT-ATP + P protocol participated in groups that processed their experiences around the topics of acculturation and parenting in a different culture, mental health, and overall systems and worldviews that impact Latine youth substance use. While the original GMIT intervention was designed to be delivered in five 30-min sessions [32], GMIT-ATP was delivered in three 1-h meetings (i.e., meeting one covered material from sessions one and two, meeting two covered material from sessions three and four, and meeting three covered material from session five and the VFHY Other Tobacco Product (OTP) module). While the intervention was delivered in person, families assigned to the GMIT-ATP + P intervention participated in concurrent but separate parent and adolescent meetings. During the online delivery format, parent meetings were held right after the completion of the adolescent meetings, mostly due to several families sharing the same computer. This format allowed two sessions to be held on one day to accommodate the participating families' time constraints and transportation needs.

**Table 2:** Cultural enhancement of GMIT-ATP for latine youth.

Session	GMIT Goals^a^	Cultural Enhancement of GMIT	GMIT-ATP + P (Parent Groups)
1	1. To motivate students to make healthier choices by showing them that most youth their age do not use alcohol, cigarettes, or drugs.	Inquire about culturally specific beliefs Latine youth may have about substances. Substance use rates for high school and middle schoolers in VA were included	• Introduce the purpose of the group and brief introductions
	2. To help students resist outside pressure by discussing the reasons that overestimation of substance use occurs; to help them pay more attention to their surroundings and what is occurring.		• Review any logistical information (e.g., audio recording, confidentiality, respecting beliefs)
2	1. To motivate students to think about the difference between a pure drug effect on behavior and the effect of beliefs on behavior.	Replaced names of characters in various examples with names that are more common in Latine communities/countries	• Define and discuss acculturation, acculturative stress, and related issues (e.g., challenges, the immigration journey, protective factors)
	2. To increase awareness of beliefs that students may have about the effects of substances		
	3. To increase students' awareness about their own beliefs, which may contribute to the choices that they make concerning substance use.		
3	1. To motivate youth to make healthier choices by helping identify how substance use may be used to cope with negative feelings and how coping this way can lead to more problems.	Inquire about culturally relevant coping strategies as well as strategies to avoid substance use provided by their social circles	• Briefly reiterate previous points about acculturation/acculturative stress
	2. To increase students' awareness of how substance use can impact their life & relationships.		• Discuss how those themes positively or negatively impact parent-child relationships and communication
	3. To discuss alternatives to drinking, smoking, and drug use.		
4	1. To help students identify pressures to use alcohol, cigarettes or drugs that may come from within themselves (e.g., stress, feeling isolated if they don't use).	Reframe substance use in culturally relevant situations (e.g., youth reported parent's not allowing them to spend time with friends alone)	• Discuss strategies that improve communication
	2. To help students learn how to resist direct pressure to use alcohol, cigarettes, or drugs.		
	3. To help students learn how to resist indirect pressures that may come from themselves.		
5	1. To increase students' awareness about situations where substances may be present.	Some US/English language alcohol ads were replaced with Spanish language or Latin American alcohol brands (e.g., Tecate instead of Budweiser)	• Briefly reiterate previous session points
	2. To help students plan and prepare for these situations.		• Discuss how those themes can impact mental health
	3. To help students evaluate whether the plans that they make for dealing with these situations are realistic and if not, to come up with a better plan so they are ready		• Discuss mental health beliefs, access/use of services, and how mental health is talked about with their children
	4. To reinforce ways to resist different kinds of pressure in different situations.		
	Other Tobacco Products Module Goals - Virginia Foundation for Healthy Youth		
6	1. Increase awareness and knowledge of Other Tobacco Products (OTPs)	Translation from English to Spanish	• Discuss other parent-driven topics if time permits
	2. Increase awareness and knowledge of the addictive qualities of nicotine.		
	3. Increase awareness and knowledge of toxic chemicals found in OTPs.		

a See Ref. [32] for more information on protocol goals.

Occasionally, a parent/caregiver or youth group contained a few parents and youth who knew each other before enrolling in the intervention due to shared membership in various community organizations (e.g., members of the same church). To enhance study participant privacy, group facilitators allowed the participants to create and agree on a group “rules” (e.g., no sharing of interpersonal content discussed in a group with non-group members) during the first session. Hence, group facilitators did not actively address whether or not participants knew each other or interacted outside of the intervention unless the participants disclosed such familiarity first. When such information was disclosed and interpreted as possibly impacting group dynamics (e.g., parent/caregivers who did not know others in the group speaking less), group facilitators implemented *personalismo*-oriented strategies (e.g., ask the quiet member a question related to a personal detail) to encourage participation from all participants.

The last session was added to the original GMIT intervention and aimed to provide educational information about alternative or other tobacco products. The module was created by a large research university in conjunction with the Virginia Foundation for Healthy Youth and Virginia's Standards of Learning. The module contained an implementation guide for teachers or facilitators, a short pre-and post-test about information youth may or may not know about alternative tobacco products, a PowerPoint aimed at sixth to twelfth graders about nicotine-like cigarettes and electronic cigarettes (e-cigarettes), and a summary handout. The PowerPoint presentation included information on the different types of OTPs, their addictive qualities, and their toxicity level. All module materials are accessible to the public and available to download from the VFHY webpage (https://www.vfhy.org/programs/tobacco/nicotine-products-prevention-modules/).

**Follow-up Assessments.** Intervention (GMIT-ATP + P) and control families (GMIT-ATP) were assessed at baseline and followed up for post-test assessments after the intervention ended and at 3-months after program completion—this provided data on the immediate and short-term program effects. Parents were asked to complete measures regarding family dynamics once but complete a second set of the measures related to the parent-child relationship for the second child. We also implemented procedures in our prior work to retain families, including offering incentives, using a tracking form, and scheduling the following assessment immediately after completing an evaluation.

**Therapist and Fidelity Training.** The first and last co-authors trained the facilitators and the research team in the GMIT-ATP and GMIT-ATP + P curriculum and fidelity procedures. All sessions were audio-recorded to evaluate treatment fidelity and potential contamination between conditions (i.e., parenting strategies introduced in the GMIT-ATP condition). These recordings were used in weekly supervision provided by two licensed and one license eligible bilingual Latine psychologists. At the end of each session, adolescents (and parents in GMIT-ATP + P) completed brief Likert-type ratings of engagement and therapist competence included with the GMIT manual and modified to cover the new content.

A licensed psychologist also oversaw the fidelity procedures, including supervised individuals completing the systematic ratings during each session. This psychologist also ensured therapists and participants completed fidelity forms at each session and coded the Motivational Interviewing Treatment Integrity (MITI; [42]. These fidelity ratings were used as a feedback tool during supervision to enhance the MITI scores.

## 8 Statistical analysis

Hypotheses and planned analyses for all aims were pre-registered (for details, see ClinicalTrials.gov, Identifier: NCT03947177). It is important to note that the study originally planned for 4 data collection points (baseline, post-training, 3-month, and 9-month follow-ups); however, due to funding cuts, we were only able to collect three waves of data (baseline, post-training, 3-month). Original sensitivity analyses conducted using G*Power [43] indicated that the proposed analysis has a power of .80 (based on an alpha of .05, a sample size of 80, 4 waves of data, and correlations of 0.50 among the repeated measures) to detect a between-within interaction effect of *f* = 0.13, which falls slightly above a “small” effect size based on Cohen's rules of thumb [44]. To test study aims, originally planned data analyses focused on a mixture of ANCOVAs and multilevel models. However, given changes in time points assessed and for consistency, we will aim to use multilevel modeling throughout all analyses to test study aims.

Given the change in assessment timepoints and that multilevel modeling will be used throughout all analyses, we re-examined our power analysis. In a recent simulation study examining statistical power for two-level models [45] for a level 2 direct effect (i.e., a test of treatment efficacy across GMIT-ATP and GMIT-ATP + P), the researchers suggest that with 80 families at level 2 and 3 level 1 events (i.e., three assessments waves), with a medium random slope variance (τ11.std = 0.09) and power = .80, the minimum detectable effect size is 0.41.

All data will be analyzed using SPSS and Mplus Version 8.6 [46]-2022). To test the efficacy of GMIT-ATP and GMIT-ATP + P on tobacco use outcomes, all analyses will be conducted using random-effects growth modeling in multilevel models using M*plus* Version 8.6 [46]-2022). Individual variability in level-1 change in outcomes from baseline to 3-month post-training follow-up will be modeled as a function of the level-2 predictor condition (i.e., GMIT-ATP vs. GMIT-ATP + P). Two parameter estimates from the growth models will be examined to determine program efficacy: (1) condition (test of acute-phase effects) and (2) condition X time (a test of maintenance). This approach will be used for all outcomes. For testing conditional effects of treatment efficacy, procedures will be the same as outlined above. Still, proposed moderators will be included as a level 2 predictor, and cross-level interactions with treatment conditions on level 1 outcomes will be examined (following approaches suggested by Aguinis and colleagues [47]. Given that this is a pilot trial, our primary focus will be on estimating confidence intervals for effect size estimates which we hope will provide a basis for a large-scale randomized trial. Additionally, given concerns of contamination of treatment effects within families, we will account for this in our analyses by only using data from the primary child (i.e., an oldest child within the family).

## 9 Discussion

The culturally enhanced group-based motivational interviewing substance use prevention program for Latine youth was designed to minimize adolescent substance and tobacco use. Our intervention program fills a gap in the motivational interviewing treatment options, as groups specifically designed for Latines and Spanish-speakers are scarce [48]. Cultural enhancement literature has shown that tailoring interventions to cultural values, language, and specific risk factors are highly effective [49]. The GMIT-ATP and GMIT-ATP + P interventions highlight how a motivational interviewing treatment can be enhanced to serve a specific community better.

Furthermore, the GMIT-ATP was tailored by centralizing the family (e.g., *familismo* values and caregiver involvement) as part of its enhancement. Family support and involvement in substance use prevention interventions for racial/ethnic minority adolescents have proven to enhance treatment efficacy [48,50]. Some have suggested this relative efficacy is because culturally-enhanced treatment targets important factors such as acculturation stress and family acculturation gaps that contribute to the development of substance use behavior among Latine adolescents [51,52]. The GMIT-ATP, therefore, aimed to positively affect Latine adolescent substance use by recognizing aspects of family structure, functioning, and acculturation.

GMIT-ATP is also cost-effective via group format and brief, greatly contributing to Latine health disparities research. Adolescent substance abuse intervention literature also suggests group formats are more common among Latine groups [53], and family-based intervention formats rather than individual are preferred among Latines [[54], [55], [56], [57]]. Such formats may also be more aligned with Latine cultural values [58,59]; however, individually or one-person adapted family-based formats may also be as efficacious as their standard family-based counterparts if conceptualized in family terms (e.g., Brief Strategic Family Therapy; [60]. Overall, GMIT-ATP offers significant potential to prevent maladaptive health behaviors for Latine youth while simultaneously promoting substance use prevention efforts by youth's families.

### 9.1 Limitations & future direction

Despite the novelty and strengths of the GMIT intervention, there are some potential challenges. First, the Latine community in the southeastern U.S. is growing, with trends including more recently arrived immigrants [61]. This growing community has historically experienced mistrust with research entities [62], so it may take time to establish rapport and trust within this community. Due to legal status concerns, many Latine mixed-status families (i.e., a family with at least one unauthorized immigrant parent and at least one U.S. citizen child [63]; worry about participating in studies due to requirements in filling out paperwork and possible disclosure in questionnaires [64]. To address this concern, the study has obtained a certificate of confidentiality to protect participants' privacy. The Latine community can also be difficult to reach and retain in intervention research [65]. To mitigate some of these challenges, the study team will work to establish a presence in community organizations that serve predominantly Latine Spanish-speaking families, as well as build rapport with families once enrolled in the program.

Furthermore, the facilitators will be bicultural and bilingual to promote continued engagement in the intervention and awareness of cultural nuances observed in the sessions. Additionally, measures were collected via self-report and substance use measures are susceptible to recall bias. It is vital to note that whilst Latine values are of great import to the study, all Latine communities are not homogenous. That is, the Latine community is made up of many different cultural and ethnic beliefs, values, and people. Therefore, the study team has ensured that materials used in the intervention apply cross-culturally. For example, beer advertisements commonly seen in grocery stores or on television in the southeastern U.S. rely more on their current physical location than the level of acculturation. Additionally, the study team included measures of acculturation and cultural values for parents and teens at all assessment time points. Finally, given ongoing changes in COVID-19 mandates associated with social gatherings, the interventions will continue to adjust for the most appropriate intervention modality, whether in person or virtual, to promote the greatest access to youth and their families.

## 10 Conclusion

Given the rise of Latine youth, more prevention efforts can address substance and tobacco use needs. This group motivational interviewing for teens (GMIT-ATP) and parents (GMIT-ATP + P) not only enhances the treatment to include the use of ATP but offers sessions in English and Spanish by trained bilingual and bicultural Latine clinicians. Additionally, because *familismo* is a critical Latine cultural value, the GMIT-ATP + P includes a parent modality where parents also process their culturally related experiences around acculturation, parenting in different cultures, mental health, and overall systems and worldviews that impact Latine youth substance use. The GMIT-ATP and GMIT-ATP + P modalities have the potential to become a sustainable program aimed at reducing Latine substance use and especially alternative tobacco products. Findings from this study will be disseminated in Latine communities and with providers working with Latine youth and can serve as a community-based model for reducing substance and tobacco use (e.g., ATP) needs in these Latine communities.

## IRB approval of human subjects

Institutional ethics approval was received for this study [HM20014435], and informed consent was obtained. The privacy rights of human subjects were also observed.

## Authors’ contribution

**Dr. Oswaldo Moreno:** Grant Submission Co-Principal Investigator, Conceptualization, Methodology, Recruitment, Supervisor, Lead Author in Writing. **Melissa Avila**: Graduate Research Assistant, Project Coordinator, Interventionist, Recruitment, Data Collection, Writing. **Isis Garcia-Rodriguez**: Graduate Research Assistant, Project Coordinator, Interventionist, Recruitment, Data Collection, Writing. **Stephanie Romo**: Graduate Research Assistant, Interventionist, Recruitment Data Collection. **Jennifer Rodriguez:** Project Coordinator, Recruitment, Data Collection, Writing. **Cristian Matos**: Project Coordinator, Recruitment, Data Collection, Writing. **Lisa S. Fuentes**: Graduate Research Assistant, Writing. **Cindy Hernandez:** Writing. **Mayra S. Ramos**: Graduate Research Assistant, Writing. **Geovani Muñoz**: Graduate Research Assistant, Writing. **Dr. Daniel Gutierrez**: Grant Submission Co-Principal Investigator, Conceptualization, Methodology, Supervisor. **Dr. Adrian J. Bravo:** Statistician Design & Writing. **Dr. Rosalie Corona:** Grant Submission Co-Principal Investigator, Conceptualization, Methodology, Recruitment, Supervisor, Senior Author in Writing.

## Registration of clinical trials

This clinical trial was registered in ClinicalTrails.gov (Identifier: NCT03947177).

## Funding source

This study was supported by the Virginia Healthy Youth Foundation Grant [*RFP 852R009]* to Drs. Corona & Moreno at Virginia Commonwealth University & Dr. Gutierrez at The College of William & Mary.

## Declaration of competing interest

The authors declare that they have no known competing financial interests or personal relationships that could have appeared to influence the work reported in this paper.
